# An investigation of the resolution of inflammation (catabasis) in COPD

**DOI:** 10.1186/1465-9921-13-101

**Published:** 2012-11-13

**Authors:** Aina Noguera, Cristina Gomez, Rosa Faner, Borja Cosio, Ana González-Périz, Joan Clària, Angel Carvajal, Alvar Agustí

**Affiliations:** 1 Hospital Universitario Son Espases, Palma de Mallorca, Spain; 2 Fundació Investigació Sanitaria Illes Balears (FISIB), CIBER Enfermedades Respiratorias (CIBERES), Mallorca, Spain; 3 Department of Biochemistry and Molecular Genetics, Hospital Clinic, University of Barcelona, Barcelona, Spain; 4 Institut d'investigacions Biomèdiques August Pi i Sunyer (IDIBAPS), Barcelona, Spain; 5 CIBER Enfermedades Hepaticas y Digestivas (CIBEREHD), Barcelona, Spain; 6 Thorax Institute, Hospital Clinic, University of Barcelona, Barcelona, Spain; 7 Institut del Torax, Hospital Clinic, Villarroel 170 (Escalera 3, Planta 5), Barcelona, 08036, Spain

**Keywords:** Chronic bronchitis, Emphysema, Inflammation, Smoking

## Abstract

**Background:**

Chronic Obstructive Pulmonary Disease (COPD) is characterized by an enhanced inflammatory response to smoking that persists despite quitting. The resolution of inflammation (catabasis) is a complex and highly regulated process where tissue resident macrophages play a key role since they phagocytose apoptotic cells (efferocytosis), preventing their secondary necrosis and the spill-over of their pro-inflammatory cytoplasmic content, and release pro-resolution and tissue repair molecules, such as TGFβ, VEGF and HGF. Because inflammation does not resolve in COPD, we hypothesized that catabasis may be abnormal in these patients.

**Methods:**

To explore this hypothesis, we studied lung tissue samples obtained at surgery from 21 COPD patients, 22 smokers with normal spirometry and 13 non-smokers controls. In these samples we used: (*1*) immunohistochemistry to assess the expression of CD44, CD36, VEGF and TGFβ in lung macrophages; (*2*) real time PCR to determine HGF, PPARγ, TGFβ, VEGF and MMP-9 gene expression; and, (*3*) ELISA to quantify lipoxin A4, a lipid mediator of catabasis.

**Results:**

We found that current and former smokers with COPD showed: (*1*) more inflammation (higher MMP-9 expression); (*2*) reduced macrophage surface expression of CD44, a key efferocytosis receptor; and, (*3*) similar levels of TGFβ, VEGF, HGF, PPARγ, and lipoxin A4 than smokers with normal spirometry, despite the presence of inflammation and disease.

**Conclusions:**

These results identify several potential abnormalities of catabasis in patients with COPD.

## Background

Chronic obstructive pulmonary disease (COPD) is characterized by an excessive inflammatory response to inhaled particles and gases, particularly tobacco smoking [1]. Recent studies have shown that this enhanced response persists after quitting smoking [2,3]. The mechanisms explaining this observation are unknown but unremitting inflammation is likely to contribute to the progression of the disease and may limit the response to anti-inflammatory therapy.

The resolution of inflammation (so-called, “*catabasis*”) is not a passive process that simply occurs when the stimuli that generated it (i.e., smoking) disappears [4,5]. On the contrary, it is a highly regulated process [4,5] that requires the coordinated action of specific lipid mediators (lipoxins, protectins and resolvins), pro-resolution and repair proteins (such as tissue growth factor beta (TGF-β), vascular endothelial growth factor (VEGF), and hepatocyte growth factor (HGF)), anti-inflammatory nuclear receptors (such as peroxisome proliferator-activated receptors (PPAR)) and the elimination of apoptotic cells (mostly neutrophils and epithelial cells) by macrophages, a process known as “efferocytosis” [4-6]. Efferocytosis prevents secondary necrosis of apoptotic cells (thus, the liberation of their pro-inflammatory cytoplasmic content) and stimulates the active release by macrophages of TGF-β, VEGF and HGF [4-6]. CD44 and CD36 are surface macrophage receptors involved in efferocytosis [7,8].

Since inflammation persists in COPD patients despite quitting smoking [2,3], we hypothesized that catabasis might be abnormal in these patients. To explore this hypothesis, we compared the markers of catabasis discussed above in lung tissue samples obtained from COPD patients, smokers with normal spirometry and never smoker controls.

## Methods

### Study design and ethics

This is a prospective, descriptive and controlled study. Lung tissue samples were collected, processed and provided by the Lung Tissue Biobank of CIBERES ( http://www.ciberes.org), as described elsewhere [9]. They were obtained from volunteers that required lung resectional surgery because of clinical reasons, mostly lung cancer. All participants signed their consent to donate these samples for research purposes, and the Ethics Committee of the CIBERES bio-bank approved their use for the current project.

### Study subjects

The diagnosis of COPD was established according to the GOLD recommendations [1]. Exclusion criteria included the presence of diffuse pulmonary inflammation or fibrosis, the absence of tumour-free lung tissue specimens, obstruction of central bronchi due to the tumour and/or previous treatment with chemotherapy or radiotherapy. Patients with other chronic inflammatory diseases, alcoholism or receiving systemic anti-inflammatory therapies were also excluded. No patient had an upper respiratory tract infection and/or receive antibiotics perioperatively.

### Lung function

Forced spirometry was obtained in all participants according to international guidelines [10]. Reference values were those of a Mediterranean population [11].

### Pulmonary tissue sampling and processing

Lung tissue samples were always obtained from non-affected distant areas from the tumour. These samples were either: (*1*) frozen rapidly in liquid nitrogen, cut into 0.3×0.3×0.3 slices and stored at – 80°C until analysis; or (*2*) fixed in formalin for immunohistochemistry analysis, as detailed below.

### Immunohistochemistry

**Figure 1:**
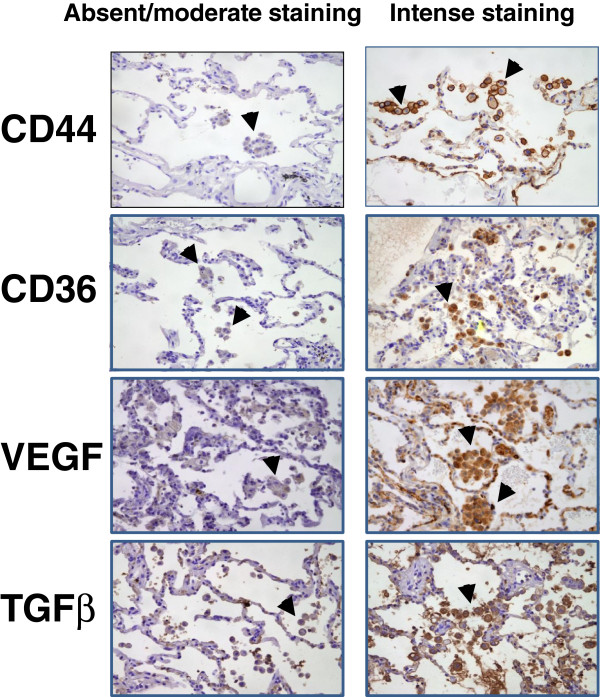
**Representative examples of different macrophage immunostaining intensities (absent/moderate vs. intense staining) for CD44, CD36, VEGF and TGFβ.** For further explanations, see text.

Formalin-fixed paraffin-embebbed tissue sections (3 μm thick) were immunostained with the following monoclonal mouse antibodies: anti-human CD44, Phagocytic Glycoprotein-1, clone DF1485 (Dako, Glostrup, Denmark); anti-human VEGF, clone VG1 (Dako, Glostrup, Denmark); anti-human TGFbeta (AbDSerotec, Oxford, UK); anti-human CD36, clone SMO, (AbDSerotec, Oxford, UK). The optimal dilutions for all antibodies were identified by examining the intensity of staining obtained with a series of dilutions, which produced specific and easily visible signals on paraffin sections made from the same control tissue before performing the staining protocol on all sections. Positive controls consisted of human tonsil tissue (CD44, VEGF), human breast carcinoma (TGFβ), and human heart tissue (CD36). Negative controls consisted of omission of the primary antibody. Sections were deparaffinized, rehydrated, and subjected to an antigen retrieval step. Endogenous peroxidase was inactivated with 0.3% hydrogen peroxide, before incubation with specific monoclonal antibodies against CD44 (1:50 dilution), CD36 (1:10), VEGF (1:50), TGFβ (1:325). LSAB + System – HRP (Dako, Glostrup, Denmark) was utilized as detection kit, and 3’- diamino-benzadine (Dako) as the chromogen. Expression of CD44, CD36, VEGF and TGFβ in five hundred macrophages was assessed in a semiquantitative analysis using a visual analogue scale. Macrophages were identified by their morphologic characteristics following previous published methodology [12,13] (Figure 1). The staining intensity was graded and expressed as: 0= absence of staining (no staining at all), 1= moderate staining (both cell membrane and cytoplasma showing mild brown staining), 2= intense staining (cell membrane showing intense, dark brown staining with or without similar cytoplasmic staining). Samples were analyzed by two expert and independent observers (AN, CG), mean values were used for analysis, and results were expressed as percentage of macrophages with intense staining.

### Gene expression

Total lung RNA was obtained from frozen tissue samples with the RNAqueous kit. RNA concentration was assessed in a Nano Drop-1000 spectrophotometer (Nano Drop Technologies Wilmington, DE) and its integrity was tested on a 6000 LabChip in a 2100 Bioanalyzer (Agilent Technologies, Palo Alto, CA). cDNA synthesis from 1 μg of total RNA was performed using the High Capacity cDNA Reverse Transcription Kit (Applied Biosystems, Foster City, CA) in a MJ Research PTC-100 thermal cycler (Bio-Rad, Hercules, CA). Validated and predesigned TaqMan Gene Expression Assays (Applied Biosystems) were used to quantify hepatocyte growth factor (HGF) (ID: Hs00300159_m1), peroxisome proliferator-activated receptor (PPAR) γ (ID: 01115513_m1), transforming growth factor (TGF) β1 (ID: Hs00171257_m1) vascular endothelial growth factor (VEGF) A (ID: Hs00173626_m1) and MMP-9 (ID: Hs00234579_m1) gene expression using β-actin (ID: Hs99999903_m1) as an endogenous control. Briefly, PCR reactions were performed in duplicate using the Universal TaqMan 2× PCR mastermix in a volume of 20 μl containing 1.25 μl cDNA. Quantitative real-time PCR amplifications were performed in an ABI Prism 7900HT Real Time PCR System (Applied Biosytems). Real time PCR results were analyzed with the Sequence Detector Software version 2.1 (Applied Biosystems). Relative quantification of gene expression was performed using the comparative Ct method. The amount of target gene, normalized to β-actin and relative to a calibrator, was determined by the arithmetic equation 2^-ΔCt^ described in the comparative Ct method (User Bulletin #2; http://docs.appliedbiosystems.com/pebiodocs/04303859.pdf).

### Lipoxin A_4_

Frozen lung tissue samples (120 mg) were homogenized in cold DPBS^++^ with an Ultra-Turrax T 25 Basic homogenizer (IKA-Werke, Staufen, Germany). Two volumes of cold methanol were added to the homogenates and incubated 30 minutes on ice. Homogenates were centrifuged at 2000 rpm for 15 minutes at 4°C and supernatants were collected. Pellets were re-suspended in 1 ml of ethanol and centrifuged again at 2000 rpm for 15 minutes at 4°C. Supernatants were combined and brought to a final volume of 10 ml with distilled water. Samples were acidified to pH 3.5, transferred into syringes and loaded onto activated C_18_-silica reverse-phase cartridges. The eluted methyl formate fraction was rapidly evaporated under a stream of nitrogen, resuspended in 1 ml of methanol and kept at −80°C until analysis of LXA_4_ by ELISA (Neogen Corporation, Lansing, MI).

### Statistical analysis

Results are presented as mean ± standard deviation, and percentages, as indicated. To compare differences between groups we used nonparametric statistics (Kruskal-Wallis test followed by Mann–Whitney U-test if appropriate). Correlations between variables of interest were explored using the Spearman correlation test. A p value lower than 0.05 (two-tailed) was considered significant.

## Results

### Characterization of participants

Table 1 presents the main demographic, clinical and functional characteristics of participants. Age was similar in all groups except that current smokers with normal spirometry were slightly younger. Most participants were males, except for never smokers where females predominate. Cumulative smoking exposure was similar between all smoker groups. All former smokers quit smoking more than 5 years before tissue sampling. As expected, spirometry was abnormal in patients with COPD and within the normal range in the other two groups. Four COPD patients had GOLD grade I and 17 GOLD grade II airflow limitation.

**Table 1:** Clinical and lung function data (mean±SD) of all participants

	**COPD patients**		**Smokers with normal spirometry**		**Non smokers**	**Overall p value**
	**Current smokers**	**Former smokers**	**Current smokers**	**Former smokers**		
N	10	11	11	11	13	
Age, years	64 ± 9	69 ± 10^*^	55 ± 8^+^	66 ± 10	62 ± 9	<0.01
Males, n (%)	10 (100)	10 (91)	8 (73)	9 (82)	3 (23)	<0.0001
Smoking exposure, pack-yr.	64 ± 41	58 ± 22	47 ± 26	64 ± 13	_	NS
FEV_1_ / FVC_1_, %	56 ± 12^&^	54 ± 7^&^	77 ± 7	77 ± 6	83 ± 4	<0.0001
FEV_1,_ % reference	70 ± 12^&^	67 ± 11^&^	97 ± 14	96 ± 19	98 ± 11	<0.0001

NS: non-significant. * p <0.01 vs. current smokers; + p < 0.05 vs. former smokers; & p<0.001 vs. current smokers, former smokers and never smokers.

### Immunohistochemistry

**Figure 2:**
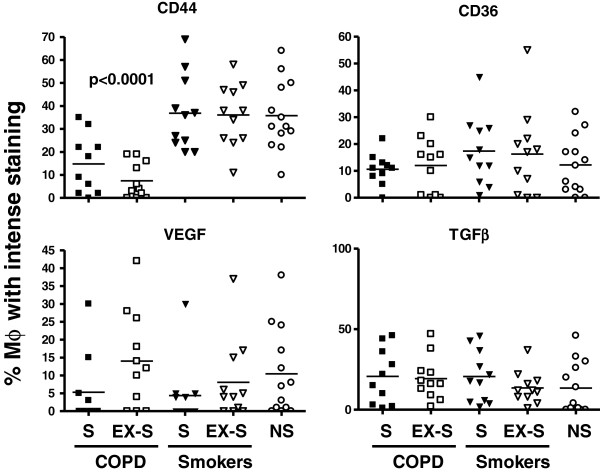
**Individual and mean (bars) values of the proportion of macrophages with intense staining for CD44 , CD36, VEGF and TGFβ in patients with COPD, smokers with normal spirometry and non-smokers.** (S = current smokers; EX-S = former smokers; NS = non-smokers). For further explanations, see text.

The pattern of macrophage immunostaining was predominantly membranous for anti-CD44 and anti-CD36 antibodies, whereas it was predominantly cytoplasmic for anti-VEGF and anti-TGFβ (Figure 1), as expected because of their respective cellular localization. Figure 2 presents the individual and mean (bars) values of the proportion of macrophages with intense immunostaining for CD44, CD36, VEGF and TGFβ in all participants. Independently of their smoking status (current vs. former), CD44 expression was lower in patients with COPD than in smokers and ex-smokers with normal spirometry and non-smokers (p<0.0001); by contrast, macrophage immunostaining of CD36, VEGF and TGFβ was similar in all groups (Figure 2). CD44 expression was not related to airflow limitation or treatment with inhaled steroids.

### Lung tissue gene expression

**Figure 3:**
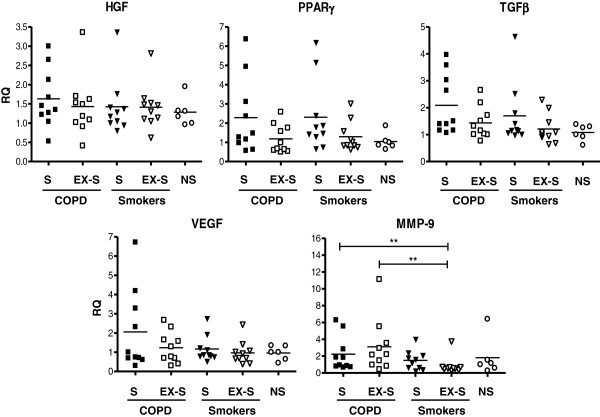
**Individual and mean (bars) values of HGF, PPARγ, TGFβ, VEGF and MMP9 gene expression in lung tissue specimens, in patients with COPD, smokers with normal spirometry and non-smokers.** (S = current smokers; EX-S = former smokers; NS = non-smokers) ** p < 0.01. For further explanations, see text.

**Figure 4:**
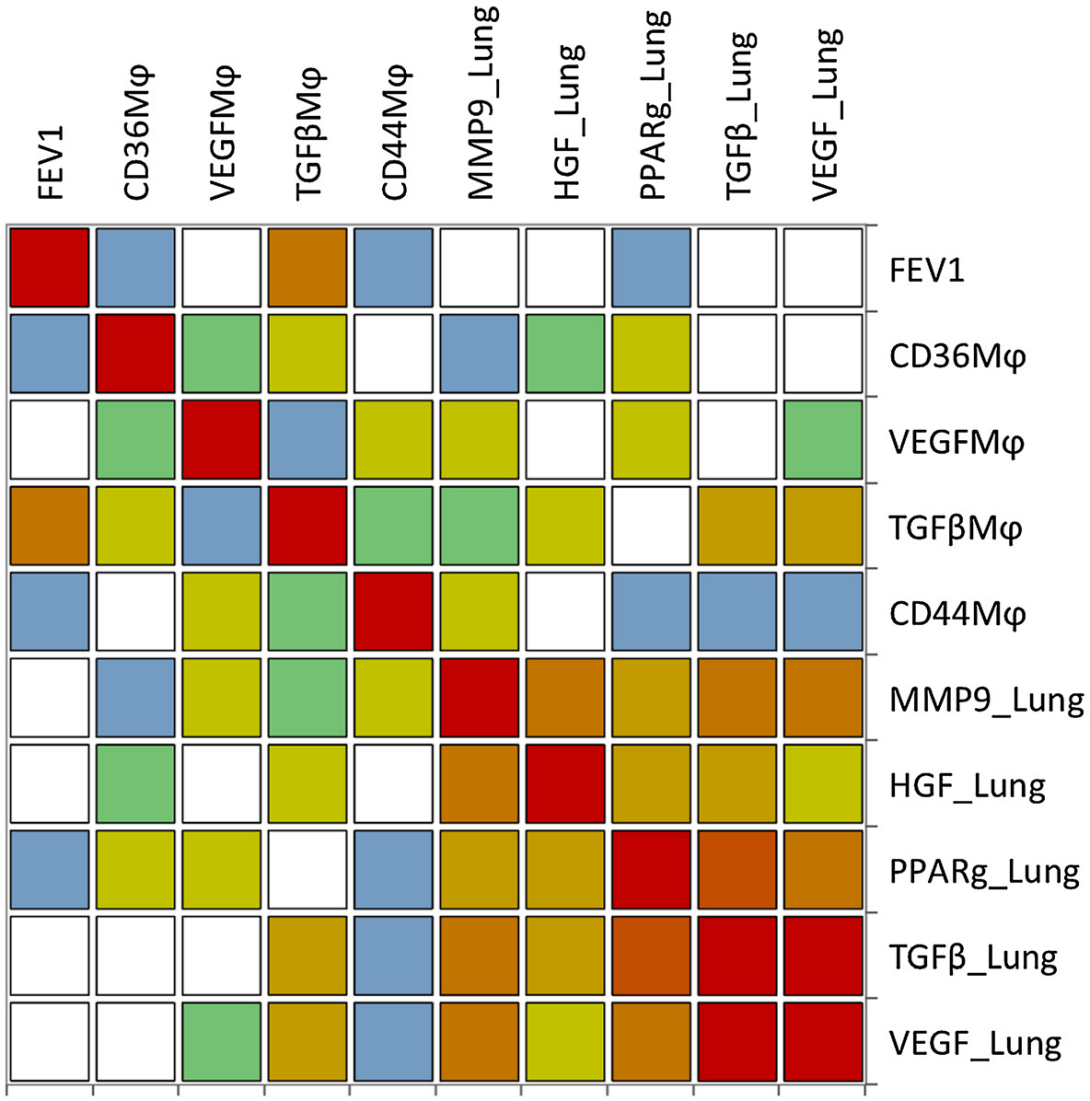
**Spearman correlations heat map.** Colours in each cell indicate the strength of the relationship, from white (no relationship) to dark red (strong relationship). The exact Rho correlation coefficient and corresponding p values are presented in Additional file 1: Table S1 (on-line supplement). For further explanations, see text.

Figure 3 presents the individual and mean (bars) values of HGF, PPARγ, TGFβ and VEGF expression in lung tissue samples. We did not observe significant differences between groups for any of these markers. Figure 3 also shows that the lung expression of MMP-9, a marker of tissue inflammation was, as expected, significantly higher in patients with COPD, both current and former smokers, than in controls. Additional file 1: Table S1 (on-line supplement) presents a correlation matrix between HGF, PPARγ, TGFβ, VEGF and MMP-9 expression in lung tissue, and the percentage of macrophages with intense staining of CD36, VEGF, TGFβ, and CD44, as well as FEV1 values in COPD patients. The same information is presented graphically (heat-map) in Figure 4 whereas Additional file 1: Table S2 (on-line supplement) presents the individual Z-scores en each patient for each inflammatory marker determined in pulmonary macrophages (M) and lung tissue extracts. It is of note that lung tissue markers (Figure 4, bottom right corner of the heat-map) were much better correlated among them than with macrophage markers, which were not particularly well correlated either among themselves (Figure 4, top left corner of the heat-map; see also Additional file 1: Table S1 on-line supplement).

### Pulmonary concentration of Lipoxin A4

**Figure 5:**
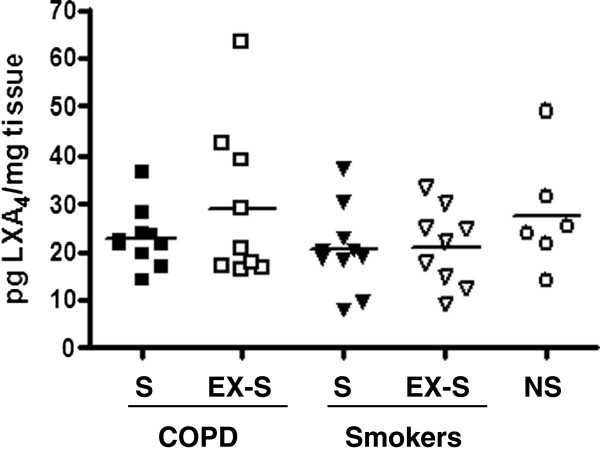
**Individual and mean (bars) values of lipoxin A4 (LXA4) concentration in lung tissue homogenates in patients with COPD, smokers with normal spirometry and non-smokers (S = smokers; EX-S = ex-smokers; NS = non-smokers).** For further explanations, see text.

The concentration of lipoxin A4 in lung tissue homogenates was similar in all groups studied (Figure 5).

## Discussion

Main results show that, compared with controls, COPD patients present: (*1*) reduced surface expression of CD44, a key macrophage efferocytosis receptor; and, (*2*) similar lung tissue levels of TGFβ, VEGF, and HGF, despite the presence of more inflammation and tissue damage.

### Previous studies

Hodge *et al*. reported that the ability of alveolar macrophages to ingest apoptotic bronchial epithelial cells and neutrophils (efferocytosis) was reduced in COPD [14,15]. We did not studied efferocytosis directly, but we found a marked reduction in the surface expression of CD44 in pulmonary macrophages (Figure 2), as other authors and our own group have previously reported in broncho-alveolar lavage fluid macrophages [14,16] . This was not the case for CD36, also in keeping with previous results [17]. Likewise, we did not find significant differences in HGF, VEGF, TGFβ or PPARγ expression in the lungs of COPD and controls (Figure 3) as others showed before [18-20]. Finally, several lipid molecules, such as lipoxins, protectins and resolvins, are also important mediators of catabasis. Lipoxins reduce neutrophil recruitment and stimulate efferocytosis [21], whereas protectins and resolvins interact with lipoxins to enhance catabasis [22]. Reduced levels of lipoxin A [23,24] and protectin D1 [25] have been reported in patients with severe asthma [26]. In COPD, results are not consistent and seem to vary according to the specific tissue studied. Vachier *et al*. found moderately increased levels of lipoxin A4 in sputum of COPD patients [27] whereas Fritscher *et al*. reported reduced concentrations in exhaled breath condensate of these patients [28]. On the other hand, in peripheral blood, Bozinovski *et al*. showed that serum amyloid A (SAA) increased disproportionately relative to LXA4 during acute exacerbation of the disease [29]. SAA is a hepatic acute-phase protein that can interact with Lipoxin A4 receptors (ALX/FPR2) and, at variance to Lipoxin A4, stimulate neutrophil recruitment and activation [29]. Interestingly, ALX/FPR2 is also important in the process of apoptotic cell clearance (efferocytosis) [30], a key component of catabasis [4-6]. In our study, in lung tissue homogenates, we did not find significant differences in the concentration of Lipoxin A4 among the five groups of subjects studied here (Figure 5).

### Interpretation of findings

A key observation of our study is that patients with COPD show a reduced surface expression of CD44 in lung macrophages (Figure 2). CD44 plays a pivotal role in efferocytosis and, hence, in the regulation of catabasis [8,31]. This is clearly illustrated in CD44 knock-out mice which, following lung injury, succumb to unremitting inflammation characterized by impaired clearance of apoptotic neutrophils and defective TGFβ release, a phenotype that could be reversed partially by reconstitution with CD44+ cells [31]. Hence, the reduced surface expression of CD44 in lung macrophages of COPD observed in our study (Figure 2) has the potential to favour secondary necrosis of apoptotic cells (known to be increased in COPD [32,33]) and to prevent an adequate pro-resolution response (enhanced TGFβ, VEGF, and HGF macrophage release). We propose that the absence of significant differences between COPD patients and controls in either protein (Figure 3) or lipid (Figure 4) mediators of catabasis actually supports a blunted catabatic response in COPD because the presence of chronic inflammation and lung tissue damage should enhance it [34]. We accept that this is a hypothesis that will have to be confirmed or refuted in future studies but, in keeping with this proposal, our group previously showed that alveolar macrophages of COPD patients release less TGFβ than controls when stimulated *in vitro* with LPS [35]. This also suggests a defective response to a well-characterized pro-inflammatory stimulus. We hypothesized that all these mechanisms can contribute to the enhanced and persistent inflammation (higher MMP-9) that characterizes COPD and to defective tissue repair. Further, a self-sustained loop can develop because MMP-9 is known to participate in the shedding of CD44 [36], although we did not find a significant relationship between MMP-9 expression and % CD44 positive macrophages in our patients (Figure 4, Additional file 1: Table S1 on-line supplement), and the absence of VEGF stimulation causes lung cell apoptosis [37]. The iteration of this loop can contribute, in combination with the absence of a significant up-regulation of PPARγ or lipoxin A4, to the presence of unremitting inflammation in COPD and disease progression, even after quitting smoking. These potential mechanisms deserve further research.

Not all smokers develop COPD. Our results show that CD44 surface expression in macrophage is only reduced in those with COPD. Why is this the case cannot be answered from our study and also requires further research. However, potential mechanisms may include one or more of the following, among others. Susceptible smokers with COPD are likely to have a genetic background that favours an enhanced inflammatory response [38], and this may initiate the process by shedding macrophage CD44 [36], hence limiting the efficiency of efferocytosis [7,8]. Alternatively, there may be individual differences in the expression of CD44 [39], making smokers with low constitutive CD44 expression more susceptible to the development of COPD since, as discussed above, CD44-deficient mice succumb to unremitting inflammation after lung injury with impaired clearance of apoptotic neutrophils and impaired activation of TGFβ [31]. It is also possible that efferocytosis of apoptotic cells may be overloaded (hence secondary necrosis allowed) if the number of apoptotic cells increases significantly, as it seems to be the case in COPD [32,33]. And, of course, none of these mechanisms is mutually exclusive, so a combination of them may also occur.

### Strengths and limitations

The fact that we studied lung tissue macrophages is at variance with previous studies investigating broncho-alveolar lavage samples and is a clear strength of our study. However, we acknowledge that our study has limitations that deserve comment. First, its descriptive nature does not allow us to make firm mechanistic inferences. Second, we did not study the expression of LXA4 receptor (ALX/FPR2) which, according to recent publications, may play an important role in the regulation of catabasis [29,30]. Third, lung tissue samples were obtained at surgery from patients suffering from cancer. Despite that samples were always obtained from non-affected distant areas from the tumour and that no patient had received radio- or chemo-therapy before surgery, we cannot exclude an influence of the tumour itself on the results of our study. However, both COPD and controls had cancer, so any eventual influence of the tumour should have influence results in both groups. Besides, this is a limitation of basically any study using human lung tissue samples. Finally, we acknowledge the relatively small sample size of our study and the fact that, due to the origin of the tissue samples, we could not study patients with more severe airflow limitation.

## Conclusions

The results of this study support the hypothesis that catabasis might be abnormal in COPD. If so, this may be a key mechanism explaining the persistence of inflammation in these patients, even after quitting smoking, and, potentially, the progression of the disease. The confirmation of this hypothesis requires further research.

## Abbreviations

COPD: Chronic obstructive pulmonary disease; TGF-β: Tissue growth factor beta; VEGF: Vascular endothelial growth factor; HGF: And hepatocyte growth factor; PPARγ: Peroxisome proliferator-activated receptor gamma; MMP-9: Matrix metalloprotease 9; PCR: Polymerase chain reaction; ELISA: Enzyme linked immunosorbent assay; LPXA_4_: Lipoxin A_4_; DPBS: Dulbecco’s phosphate buffered saline; SSA: Serum amyloid A.

## Competing interests

Authors declare that they have no competing interests.

## Authors' contributions

AN contributed to the tissue processing, immunohistochemistry assays, planning and designing the study, and writing and revision of the manuscript. CG contributed to the tissue processing, immunohistochemistry assays, planning and designing the study, and writing and revision of the manuscript. RF contributed to guiding the gene expression studies, planning and designing the study, and writing and revision of the manuscript. BC contributed coordinating the patient/tissue selection process and revision of the manuscript. AGP carried out the molecular genetic studies and the immunoassays, and contributes to revision of the manuscript. JC contributed to the gene expression studies and immunoassays, and revision of the manuscript. AC contributed to collecting the lung tissues and revision of the manuscript. AA contributed to designing and supervising the study to its completion, and the writing and revision of the manuscript. All authors read and approved the final manuscript.

## Supplementary Material

Additional file 1 **Table S1.** Spearman Rho correlation matrix (A) and corresponding p values (B) for all biomarkers determined in pulmonary macrophages (M) and lung tissue extracts, as depicted graphically in heat-map shown in Figure 4. For further explanations, see text. Table S2. Z-scores of individual patients for each inflammatory marker determined in pulmonary macrophages (M) and lung tissue extracts. For further explanations, see text. Click here for file
