# Supplementary material for: An investigation of the resolution of inflammation (catabasis) in COPD
Source: Respir Res. 2012 Nov 13;13(1):101. doi: 10.1186/1465-9921-13-101 (PMC3546860; doi:10.1186/1465-9921-13-101)
Supplement: Additional file 1 — Table S1. Spearman Rho correlation matrix (A) and corresponding p values (B) for all biomarkers determined in pulmonary macrophages (M) and lung tissue extracts, as depicted graphically in heat-map shown in Figure 4. For further explanations, see text. Table S2. Z-scores of individual patients for each inflammatory marker determined in pulmonary macrophages (M) and lung tissue extracts. For further explanations, see text. [file 1465-9921-13-101-S1.doc]

29/08/2026

**ON-LINE SUPPLEMENT**

**AN INVESTIGATION OF THE RESOLUTION OF INFLAMMATION (CATABASIS) IN COPD**

Aina Noguera* ^1,2^, Cristina Gomez* ^1^, Rosa Faner*^2^, Borja Cosio^1,2^, Ana González-Périz^3,4,5^, Joan Clària^3,4,5^, Angel Carvajal^1^, Alvar Agustí^2,4,6^.

**Table 1 (on-line supplement)**. Spearman Rho correlation matrix (A) and corresponding p values (B) for all biomarkers determined in pulmonary macrophages (Mϕ) and lung tissue extracts, as depicted graphically in heat-map shown in Figure 4. For further explanations, see text.

| **(A)** | | |  |  |  |  |  |  |  |  |
| --- | --- | --- | --- | --- | --- | --- | --- | --- | --- | --- |
|  |  |  |  |  |  |  |  |  |  |  |
| Variables | FEV1 | CD36MΦ | VEGFMΦ | TGFβMΦ | CD44MΦ | MMP9_Lung | HGF_Lung | PPARg_Lung | TGFβ_Lung | VEGF_Lung |
| FEV1 | **1** | -0.257 | -0.060 | 0.429 | -0.205 | -0.051 | -0.022 | -0.317 | 0.049 | 0.053 |
| CD36MΦ | -0.257 | **1** | -0.134 | 0.186 | 0.077 | -0.310 | -0.191 | 0.182 | 0.086 | -0.068 |
| VEGFMΦ | -0.060 | -0.134 | **1** | -0.224 | 0.117 | 0.178 | 0.009 | 0.191 | 0.036 | -0.167 |
| TGFβMΦ | 0.429 | 0.186 | -0.224 | **1** | -0.114 | -0.135 | 0.186 | 0.004 | 0.313 | 0.329 |
| CD44MΦ | -0.205 | 0.077 | 0.117 | -0.114 | **1** | 0.130 | -0.052 | -0.212 | -0.342 | -0.309 |
| MMP9_Lung | -0.051 | -0.310 | 0.178 | -0.135 | 0.130 | **1** | 0.405 | 0.313 | 0.441 | 0.435 |
| HGF_Lung | -0.022 | -0.191 | 0.009 | 0.186 | -0.052 | 0.405 | **1** | 0.353 | 0.292 | 0.177 |
| PPARg_Lung | -0.317 | 0.182 | 0.191 | 0.004 | -0.212 | 0.313 | 0.353 | **1** | **0.701** | **0.597** |
| TGFβ_Lung | 0.049 | 0.086 | 0.036 | 0.313 | -0.342 | 0.441 | 0.292 | **0.701** | **1** | **0.914** |
| VEGF_Lung | 0.053 | -0.068 | -0.167 | 0.329 | -0.309 | 0.435 | 0.177 | **0.597** | **0.914** | **1** |
| *Bold values indicate p<0.05* | | | | | | |  |  |  |  |
|  |  |  |  |  |  |  |  |  |  |  |

| **(B)** |  |  |  |  |  |  |  |  |  |  |
| --- | --- | --- | --- | --- | --- | --- | --- | --- | --- | --- |
|  |  |  |  |  |  |  |  |  |  |  |
| Variables | FEV1 | CD36MΦ | VEGFMΦ | TGFβMΦ | CD44MΦ | MMP9_Lung | HGF_Lung | PPARg_Lung | TGFβ_Lung | VEGF_Lung |
| FEV1 | **3.92367E-06** | 0.259 | 0.795 | 0.053 | 0.370 | 0.831 | 0.929 | 0.174 | 0.836 | 0.823 |
| CD36MΦ | 0.259 | **0** | 0.562 | 0.416 | 0.737 | 0.183 | 0.417 | 0.440 | 0.716 | 0.777 |
| VEGFMΦ | 0.795 | 0.562 | **0** | 0.328 | 0.612 | 0.449 | 0.968 | 0.417 | 0.878 | 0.480 |
| TGFβMΦ | 0.053 | 0.416 | 0.328 | **0** | 0.623 | 0.568 | 0.429 | 0.987 | 0.178 | 0.156 |
| CD44MΦ | 0.370 | 0.737 | 0.612 | 0.623 | **0** | 0.580 | 0.828 | 0.368 | 0.141 | 0.184 |
| MMP9_Lung | 0.831 | 0.183 | 0.449 | 0.568 | 0.580 | **0** | 0.077 | 0.178 | 0.053 | 0.056 |
| HGF_Lung | 0.929 | 0.417 | 0.968 | 0.429 | 0.828 | 0.077 | **0** | 0.126 | 0.210 | 0.451 |
| PPARg_Lung | 0.174 | 0.440 | 0.417 | 0.987 | 0.368 | 0.178 | 0.126 | **0** | **0.001** | **0.006** |
| TGFβ_Lung | 0.836 | 0.716 | 0.878 | 0.178 | 0.141 | 0.053 | 0.210 | **0.001** | **0** | **< 0.0001** |
| VEGF_Lung | 0.823 | 0.777 | 0.480 | 0.156 | 0.184 | 0.056 | 0.451 | **0.006** | **< 0.0001** | **0** |
| *Bold values indicate p<0.05* | | | | | | |  |  |  |  |

**Table 2 (on-line supplement)**. Z-scores of individual patients for each inflammatory marker determined in pulmonary macrophages (Mϕ) and lung tissue extracts. For further explanations, see text.

| **Patient** | **1** | **2** | **3** | **4** | **5** | **6** | **7** | **8** | **9** | **10** | **11** | **12** | **13** | **14** | **15** | **16** | **17** | **18** | **19** | **20** | **21** |
| --- | --- | --- | --- | --- | --- | --- | --- | --- | --- | --- | --- | --- | --- | --- | --- | --- | --- | --- | --- | --- | --- |
| FEV1 | -1,44 | -1,18 | -1,09 | -1,01 | -0,83 | -0,75 | -0,57 | -0,48 | -0,48 | -0,48 | -0,40 | -0,14 | 0,21 | 0,39 | 0,39 | 0,82 | 0,82 | 1,17 | 1,34 | 1,69 | 2,04 |
| CD36Mϕ | 1,03 | 1,39 | -0,40 | 1,27 | 0,44 | -0,04 | 0,20 | -1,35 | -1,35 | 0,56 | -1,35 | -0,16 | -1,23 | -0,76 | 2,23 | -0,28 | 0,56 | 0,44 | 0,08 | -0,04 | -1,23 |
| VEGFMϕ | 0,01 | 0,17 | 0,41 | -0,55 | 1,45 | -0,79 | -0,79 | 1,29 | -0,79 | -0,79 | -0,79 | -0,47 | 2,57 | -0,79 | -0,79 | 1,61 | 0,65 | -0,79 | -0,79 | -0,39 | 0,33 |
| TGFβMϕ | -0,26 | -0,73 | -0,33 | 0,54 | -0,93 | -1,27 | -1,20 | -1,20 | -0,13 | 1,81 | -0,67 | 0,20 | -0,46 | 0,14 | 0,27 | 1,07 | -0,26 | 1,61 | 1,74 | -1,13 | 1,21 |
| CD44Mϕ | 0,56 | 0,11 | -0,34 | 0,83 | -0,88 | 2,00 | -0,97 | 0,29 | -0,97 | -0,52 | -0,52 | 1,82 | 0,02 | -0,97 | -0,61 | 1,73 | -1,15 | -0,61 | 0,47 | 0,83 | -1,15 |
| MMP9_Lung | -0,69 | -0,71 | 0,33 | 3,18 | 1,08 | -0,45 | -0,20 | 0,06 | -0,66 | -0,32 | -0,18 | -0,31 | -1,01 | -0,64 | -0,74 | -0,70 | -0,75 | -0,84 | 0,10 | 1,07 | 1,36 |
| HGF_Lung | 0,34 | -0,51 | -1,03 | 0,24 | 0,52 | -0,43 | 0,01 | -0,33 | 0,35 | -0,52 | -0,17 | -0,05 | -1,72 | -0,41 | -0,42 | -0,03 | -1,08 | -0,74 | 2,27 | -0,91 | 2,91 |
| PPARg_Lung | 1,33 | 1,14 | 1,04 | -0,31 | 1,83 | -1,07 | -0,45 | -0,78 | -0,97 | -0,57 | -0,19 | 0,70 | -1,68 | 1,05 | 0,04 | -1,37 | -0,63 | -1,32 | 0,12 | -0,89 | 1,31 |
| TGFβ_Lung | -1,10 | 0,22 | 0,75 | 0,19 | 1,11 | -1,64 | -0,08 | -0,23 | -0,80 | 0,92 | 0,01 | 0,57 | -2,70 | 0,31 | 1,09 | -1,25 | -0,35 | -1,60 | 0,97 | -1,04 | 1,95 |
| VEGF_Lung | -0,88 | -0,47 | 2,38 | 0,11 | 0,15 | -1,04 | -0,18 | -0,64 | -0,59 | 0,27 | 0,22 | 0,24 | -1,34 | 0,17 | 1,25 | -1,15 | -0,81 | -0,98 | 0,58 | -0,81 | 2,17 |
